# Development of Dyslexia: The Delayed Neural Commitment Framework

**DOI:** 10.3389/fnbeh.2019.00112

**Published:** 2019-05-21

**Authors:** Roderick I. Nicolson, Angela J. Fawcett

**Affiliations:** ^1^Department of Psychology, Edge Hill University, Ormskirk, United Kingdom; ^2^Department of Psychology, College of Human and Health Sciences, Swansea University, Swansea, United Kingdom

**Keywords:** dyslexia, cerebellum, procedural learning, functional networks, executive function, neural commitment, language

## Abstract

It is now evident that explanations of many developmental disorders need to include a network perspective. In earlier work, we proposed that developmental dyslexia (DD) is well-characterized in terms of impaired procedural learning within the language networks, with the cerebellum being the key structure involved. Here, we deepen the analysis to include the child’s developmental process of constructing these networks. The “Delayed Neural Commitment (DNC)” framework proposes that, in addition to slower skill acquisition, dyslexic children take longer to build (and to rebuild) the neural networks that underpin the acquisition of reading. The framework provides an important link backwards in time to the development of executive function networks and the earlier development of networks for language and speech. It is consistent with many theories of dyslexia while providing fruitful suggestions for further research at the genetic, brain, cognitive and behavioral levels of explanation. It also has significant implications for assessment and teaching.

## Introduction

Developmental dyslexia (DD) is traditionally defined as “*a disorder in children who, despite conventional classroom experience, fail to attain the language skills of reading, writing and spelling commensurate with their intellectual abilities*” (World Federation of Neurology, 1968). Many attempts have been made to provide fuller or more theory-based definitions of dyslexia, but none has proved as enduring as this initial definition. Very extensive research has taken place over the past three decades, but progress toward a clear understanding, a clear diagnostic system or an effective support system remains elusive.

A critical problem for studying dyslexia is that by the time dyslexia is identified—or even suspected—a child will already be at least 5 years and probably considerably older, and therefore his or her developmental history is lost to detailed investigation. It is now established that the brain’s primary network structures are developed within the first 2 years of life, both for white matter structural connectivity, and for functional networks including the default mode network, the dorsal attention network and the salience network (Gilmore et al., 2018), and consequently, the early childhood period may prove critical for the understanding of the development of dyslexia.

The need for a developmental analysis of dyslexia is well-established (Karmiloff-Smith, 1998; Goswami, 2003). There have been two major European longitudinal studies of children born to dyslexic parents (Lyytinen et al., 2004; van der Leij et al., 2013), but these have the inevitable limitations of atypical samples (owing to the need for familial incidence), delay between study design and dyslexia diagnosis (hence the tests undertaken on infants may be outdated by study end) and moreover, it is likely that the parents of participants will be alert to any dyslexia-like issues and may take additional actions. In short, longitudinal studies provide additional converging evidence but cannot in themselves provide the necessary theoretical foundations.

Finally, there is now very extensive evidence that dyslexia overlaps markedly with several other learning disabilities, including Specific Language Impairment (SLI), Attention Deficit and Hyperactivity Disorder (ADHD), and Developmental Coordination Disorder (DCD; Fletcher et al., 1999; Gilger and Kaplan, 2001; Hill, 2001; Boada et al., 2012). Not surprisingly, these issues further complicate the appropriate diagnostic and assessment methods for dyslexia.

Our approach to this series of problems is to take a broader developmental perspective, starting at gestation, and making our way through the developmental processes of the first 5 years of life. We take the view that the extant theories of dyslexia provide a valuable analysis of the reading-level symptoms and that a complete framework should provide an explanation of the developmental processes that lead to this range of symptoms. The article, therefore, comprises four sections. First, we present an overview of explanatory theories for dyslexia, including our three frameworks for the explanation of dyslexia, namely automatization deficit, cerebellar deficit framework and the subsequent procedural learning framework, with the intention of highlighting potential synergies between the many theories. We then re-present three experimental studies that are interpretable only within a learning framework, and provide direct evidence of the delay in skill learning exhibited by dyslexic children. The third section attempts to link these findings and theories to the current state of the art in terms of development of language skills and neural networks, highlighting the process of neural commitment considered to underlie much of this developmental trajectory. Finally, we develop the proposal that “Delayed Neural Commitment (DNC),” not just at skill level but also at network level, provides not only a parsimonious characterization of the development of dyslexia but also unique insights into how to mitigate problems caused by this developmental difference.

### Levels of Analysis and Theories of Dyslexia

There are many theories for the causes of dyslexia. Accessible overviews of a range of theories were given in Demonet et al. (2004) and also in Nicolson and Fawcett (2008). A more recent overview focusing on the dominant phonological deficit framework is provided in Peterson and Pennington (2012). It is beyond the scope of this section to give even a summary of the individual theories, but it is valuable to list some of the more prominent approaches, since it is our intention to try to integrate them within a coherent developmental framework.

When considering theories it is useful to distinguish three levels of explanation (Morton and Frith, 1995): the behavior level (which is directly observable, such as reading), the cognitive level (in terms of underlying theoretical constructs such as memory, language and processing speed) and the brain level (which focuses on neural structures and process). More recent research suggests the need for two further levels: with the genetic level as the deepest level and the “network” level between the cognitive level and the brain level.

#### Behavioral Level

Following the standard medical model, the behavioral manifestations may be seen as symptoms of the underlying cause. The primary symptom of dyslexia is, of course, poor reading. For much dyslexia research, the focus of attention is on reading-related symptoms, and consequently, this research has tended to focus on reading and pre-reading skills. Behavior level theories could, therefore, include lack of opportunity, lack of experience, lack of letter knowledge or lack of “concepts about print” (Clay, 1993). However, broadening the scope to an attempt to understand the underlying causes brings a range of further potential symptoms into play, in much the same way as in medical diagnosis the symptoms might be fever, but in order to establish the underlying cause a range of further investigations must be made, leading to the establishment of a range of secondary symptoms that, together with the primary symptoms, allow a differential diagnosis of underlying cause. This is particularly important at the genetic level, where having an appropriate phenotype (symptom) is crucial.

#### Cognitive Level

Many theories have attempted to explain the behavioral symptom at the next level, namely the cognitive level, thereby providing a potentially causal explanation. The dominant cognitive level theory is the phonological deficit hypothesis (Stanovich, 1988). The hypothesis proposes that the reading difficulties are attributable to problems in phonological processing, that is, breaking a word down into its constituent sounds. These difficulties cause problems in sound segmentation and also in word blending, both of which are critical for the development of reading and spelling. There has been extensive research on phonological deficit. However, phonological deficit is by no means the only relevant theory. There are actually many other cognitive level theories, some narrower, some broader. We provide representative examples below. Each one of them has merit—supportive evidence and also successful remediation studies.

The double deficit hypothesis (Wolf and Bowers, 1999) identified two risk factors for reading acquisition: phonological deficit and processing speed deficit. Children who suffered from a “double deficit” were shown to have a much higher risk of reading problems than children with only one. Phonological deficit theorists argue that this is best seen as a variant of the phonological deficit hypothesis, and may attempt to subsume phonology, working memory and processing speed within their framework-“*deficits in phonological coding [underlie problems in] phonological awareness, alphabetic mapping, phonological decoding, verbal memory, and name encoding and retrieval*” (Vellutino et al., 2004, p. 31). A later theory, the phonological access theory (Ramus and Szenkovits, 2008), proposes that the phonological representations are intact but with slower and more effortful access.

The speech rhythm deficit hypothesis (Goswami, 2002) holds that the phonological problems arise from difficulties in perceiving the onset of the amplitude envelope which forms the basis of determining the prosody of an utterance (and hence identifying syllable boundaries).

The visuo-spatial attention deficit hypothesis (Facoetti et al., 2003) attributed reading-related deficits to difficulties in “covert orienting,” that is, preparing to switch attention to a new specific location while still concentrating on the current location. This is a process required for skilled reading in that the reader is covertly attending to the next words while reading the currently fixated one. A related hypothesis (Bosse et al., 2007) holds that visual attention span is reduced in dyslexia.

Further visual hypotheses relate to fixation accuracy and stability, together with saccadic accuracy. Stein and his colleagues identified eye movement differences (Eden et al., 1994), and several authors have reported disadvantages with visual crowding or advantages for reading with larger fonts (Moores et al., 2011; Schneps et al., 2013). An independent approach to auditory processing, Tallal et al. (1993) proposed that in common with children with SLI, dyslexic children have specific problems in rapid auditory processing. Both these frameworks have been interpreted at the brain level in terms of the magnocellular deficit hypothesis (see below).

Finally, two hypotheses address the learning processes in dyslexia. A series of studies by Froyen et al. (2011) strongly criticized the phonological deficit account as being a description rather than explanation, and provided evidence that dyslexic children have specific difficulties in integrating the visual letters with their sounds (that is, the visual-auditory cross-modality links are not made automatically).

This visual-auditory integration deficit hypothesis may be seen as a specific instance of the automatization deficit hypothesis (Nicolson and Fawcett, 1990), applied to the reading domain. The automatisation deficit framework proposes that dyslexic children have difficulties making any skill automatic, whether it is a cognitive skill as in reading, or a motor skill, as in balance or catching. A consequence of the incomplete automaticity is that dyslexic children need to try harder, to “consciously compensate,” even for routine skills that normally-achieving children undertake without effort.

Problems, therefore, become apparent in dual tasks or more complex tasks, where it is not possible to consciously compensate both.

#### Brain Level

Theories framed at the brain level typically attempt to explain cognitive level deficits in terms of the brain structures that cause them.

The most prevalent brain-level hypothesis for dyslexia is in terms of sensory processing and in particular the “magnocellular deficit” hypothesis. There is extensive, albeit inconsistent, evidence of specific visual problems relating to detection of low contrast moving visual gratings (Eden et al., 1996), which was attributed to impaired function in the visual magnocellular system. In an attempt to integrate both visual and auditory magnocellular approaches (Stein, 2001, 2018) has suggested that they may be a pan-sensori-motor abnormality in the magnocellular systems for audition, vision and action.

A broader brain-level theory is our cerebellar deficit hypothesis (Nicolson et al., 1995, 2001). The cerebellum is a major brain structure, containing over half the brain’s neurons (Brodal, 1981), and with two-way connections to almost all other head and body nervous systems (Bostan et al., 2013). It has a crystalline structure that supports the development of recurrent circuits (“microcomplexes”) able to scaffold the acquisition and/or execution of a range of motor skills (Ito, 1984, 2008). The advent of brain imaging highlighted the involvement of the cerebellum in cognitive skills and sensory processing, as well as language through connections to Broca’s area, thereby providing a natural link to the multiple perspectives on dyslexia. Following direct evidence of specific cerebellar deficits in a range of skills (Nicolson et al., 1995, 1999, 2002; Fawcett et al., 1996; Fawcett and Nicolson, 1999) we developed the cerebellar deficit framework for dyslexia, and argued that the framework was able to subsume all the above accounts (automatisation deficit, phonological deficit, speed deficit and sensory integration deficit) at the cognitive level, while providing a potentially causal link to the underlying brain structures and mechanisms.

**Figure 1:**
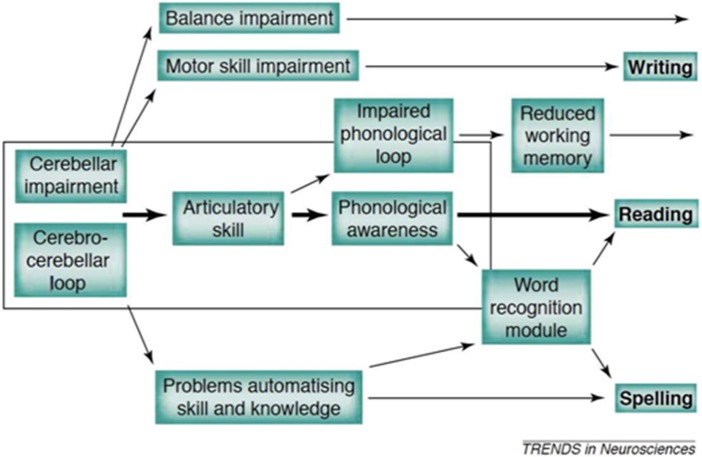
The developmental causal chain (Nicolson et al., 2001).

Of particular interest here, we created the first truly developmental account (see Figure 1) which proposed that a range of factors could be at play in the pre-reading years, and that these could (depending on the number of cerebellar networks involved) lead to a range of symptoms within and beyond reading-related skills. It may be seen from Figure 1 that the major route of impairment is *via* phonological processing (linked to speech production weaknesses), with additional problems arising from working memory limitations and also (distinctively) from automatisation problems. These problems give rise (in due course) to the problems of reading and spelling that are the defining features of dyslexia. The framework has several difficulties, with the major assessment difficulty being that it is extremely difficult to isolate the role of the cerebellum from other brain structures because it works in tandem to optimize performance. Furthermore, given its putative role in the developmental process, standard cross-sectional performance tests lack the necessary investigative power. Third, the cerebellum is a huge structure, and therefore it is critical to identify more specifically which networks are those centrally involved (Stoodley and Stein, 2013).

Fourth, a range of studies have demonstrated that whereas almost all dyslexic children show a phonological deficit, only a subset show difficulties in motor skill, and these children may show additional disorders such as DCD or ADHD (Ramus et al., 2003). Finally, once the effects of phonological deficits have been accounted for, motor skill deficits do not contribute to the reading deficits (White et al., 2006). We addressed these issues at the time (Nicolson and Fawcett, 2005, 2006) and so should not do so here. Subsequent research has clearly supported the general framework (Alvarez and Fiez, 2018). We discuss this framework and the subsequent Procedural Learning Deficit framework in the following sections.

#### The Neural Network Level

Recent research in cognitive neuroscience has made it clear that brain regions work together to create skills, and therefore it is important to introduce a level in between the cognitive level and the brain level, namely the network level. As discussed in section “The Cognitive Neuroscience of Neural Network Development” a biological neural network comprises a group of neurons that are either chemically or functionally related. Introducing this level addresses some difficulties for brain-level theories. Consider our own cerebellar deficit hypothesis (Nicolson et al., 1995, 2001). It is clear that there is impairment of performance in many skills that involve the cerebellum and indeed there is clear evidence of differences in cerebellar structure (Eckert, 2004; Pernet et al., 2009) and function (Nicolson et al., 1999; Alvarez and Fiez, 2018). However, as noted by Zeffiro and Eden (2001), it is quite possible that the cerebellum is actually functioning at normal levels, but that it is really receiving poor quality information from other brain regions such as the senses. It is, therefore, an “innocent bystander” and the true underlying cause lies elsewhere. This is in fact why we explicitly included other brain networks linked to the cerebellum in our reformulation of the hypothesis (Nicolson et al., 2001). A fuller analysis of the issues involved led directly to our third framework for dyslexia, which is the Procedural Learning Deficit hypothesis (Nicolson and Fawcett, 2007).

The distinction between procedural and declarative systems is long-established in cognitive neuroscience (Squire et al., 1993). In our research, we had established a range of procedural problems for dyslexia but no declarative problems. Interestingly, a novel analysis by Ullman (2004) highlighted the fact that there are also procedural and declarative systems for language, with the procedural system corresponding to the “mental grammar.” Ullman and Pierpont (2005) claimed that SLI could be attributed to the abnormal function of the cortico-striatal branch of the language-based procedural memory system.

In order to highlight the developmental aspects, we adopted the terminology “procedural learning system” and proposed that dyslexia could be assigned to the cerebellar branch of the language-based procedural learning system. This eliminated (or at least finessed) the issue of which aspects of the network were actually the “culprit” and which the “bystander.”

There is strong, recent evidence for the framework, which is consistent with both automatisation deficit and cerebellar deficit (and provides a natural account of the phonological deficits). For more specific evidence for the network analysis, serial reaction time studies (procedural learning) show a consistent deficit for dyslexia, coupled with consistent problems in procedural learning (Lum et al., 2013). Deficits in consolidation of procedural skill learning in dyslexic students have also been found (Nicolson et al., 2010). Interestingly, there is also a greater impact on the procedural learning of letters than motor sequences (Gabay et al., 2012). Most intriguingly, a study has demonstrated better performance for dyslexic children than age-matched controls for learning and retention of declarative memory (Hedenius et al., 2013).

It is important, however, to acknowledge that more recent research has revealed the existence of many more neural networks than originally identified, as we discuss in the section “The Cognitive Neuroscience of Neural Network Development.”

#### Genetic Level

There is clear evidence of genetic transmission of dyslexia—a male child with dyslexic parent or sibling has a 50% chance of being dyslexic (Pennington et al., 1991). There has been very extensive genetic research over the past 15 years, and genetic theories have identified a range of genes, many of which are involved in neuronal migration. Unfortunately, there has been a disappointing lack of progress, which contrasts markedly with the transformation in genetics techniques over that period, and the extensive research that has taken place (Carrion-Castillo et al., 2013; Becker et al., 2014). A key difficulty is that genetic analyses cannot perform any better than the phenotypes (behavioral manifestations) collected, and given that reading difficulty is too diffuse a symptom, an appropriate phenotype or endophenotype is dependent on the quality of the theoretical framework investigated. In short, genetic analyses are best suited to providing converging evidence relating to current theories, rather than directing the development of new theories.

### The Cognitive Neuroscience of Neural Network Development

A major development in brain imaging research in the past decade has been the development of the tools to investigate structure and function at the network level. In particular, Diffusion Tensor Imaging (DTI) allowed the identification of white matter tracts and, in parallel, analysis of functional synchrony over time facilitated the identification of intrinsic connectivity networks. Initial research led to the identification of the “Default Network (or Default Mode Network; Buckner et al., 2008), which is engaged when a person is not actively doing anything, and can be involved in thinking about others, thinking about themselves, remembering the past, and planning for the future. Subsequent research (Yeo et al., 2011) highlighted a further six networks: the somatomotor network relates to the body and to motor coordination. The dorsal attentional network is thought to mediate the top-down guided voluntary allocation of attention to locations or features (Vossel et al., 2014). The ventral attentional network is alternatively termed the Cingulo-Opercular network, and is often labeled the salience network. The fronto-parietal network seems to initiate and adjust control; the cingulo-opercular component provides stable ‘set-maintenance’ over entire task epochs” (Dosenbach et al., 2008). Early research limited network analysis to the cerebral cortex, but subsequent research established that the cerebellum was involved in all seven networks (Buckner et al., 2011) “*Quantitative analysis of 17 distinct cerebral networks revealed that the extent of the cerebellum dedicated to each network is proportional to the network’s extent in the cerebrum with a few exceptions, including primary visual cortex, which is not represented in the cerebellum*.” A valuable overview of the power of functional connectivity analyses in the case of autism spectrum disorder is provided by D’Mello and Stoodley (2015).

Converging evidence regarding the structural linkage at the systems level between key brain regions, namely the frontal cortex, the basal ganglia and the cerebellum has also recently emerged (Caligiore et al., 2017).

More recent analyses have investigated how these functional networks develop with maturation. A clear review of developments in early childhood is provided in Gilmore et al. (2018), who conclude (p. 134) that “*Studies to date have found that, by birth, major white-matter tracts are in place and white-matter structural networks and sensorimotor resting-state functional networks are well developed. The first year of life is a period of robust gray-matter growth, rapid myelination and maturation of the microstructure of existing white-matter tracts and development of higher-order resting-state functional networks. By age* 2 *years, the fundamental structural and functional architecture of the brain seem to be in place, and the brain maturation that occurs in later childhood is much slower*.” A recent study of development of attentional networks is provided by Rohr et al. (2018). In the case of reading, it is well known that the neural circuits involved show major structural changes with expertise, leading to the integration of the “Visual Word Form Area” (VWFA) into the initial circuitry (Schlaggar and McCandliss, 2007; Ben-Shachar et al., 2011). Naturally, given the lack of reading fluency for dyslexic children, there are clear differences in VWFA connectivity and function (van der Mark et al., 2009, 2011; Koyama et al., 2013; Finn et al., 2014; Schurz et al., 2015). Unfortunately, it is not clear why these differences arise and how best to facilitate development of efficient connectivity in these cases.

In summary, the major recent development in cognitive neuroscience has been the identification of a range of neural networks that develop in early childhood. Unfortunately, almost all explanatory theories for dyslexia predate these insights, and so we now have the opportunity to revisit these theories in the light of these recent developments. We propose that the framework of learning and network development provides unique insights into the development of dyslexia, and indeed opportunities for dyslexia support, as we discuss below.

## Three Studies of Development of Dyslexia

Before proceeding to our developmental analysis, it is important to provide some empirical data that have helped guide our subsequent theoretical approach but are perhaps not as well integrated into the literature as other studies. The results present a severe—indeed insuperable—challenge to most of the theoretical approaches outlined above.

In all studies reported here, full written informed consent was obtained from parents/guardians for participation in the research and participants were informed that they could withdraw at any time. Full ethics permission for all studies was obtained from the University of Sheffield ethical committee.

### Study 1: Skill, Development and Dyslexia

In our literature reviews around 1990, we were struck by the fact that each research group provided convincing evidence that dyslexic children were impaired on the tests that they administered but neglected to test for deficits outside their own field. We, therefore, developed a cross-sectional research design that included six groups of children—three groups of dyslexic children at ages 8, 11 and 15 years, together with three groups of normally achieving children matched for age and IQ. Furthermore, the two older groups of dyslexic children were also matched for reading age with the two younger groups of controls (Dys 15 with Cont 11, Dys 11 with Cont 8). This design allows a number of different analyses to be performed and provides a method of investigating the effects of maturation on the skills involved. In addition to psychometric tests of IQ and literacy, four types of test were used, designed to offer no opportunity for “conscious compensation” and tapping the range of skills affected in dyslexia—phonological skill, working memory, information processing speed, and motor skill (Nicolson and Fawcett, 1995).

**Figure 2:**
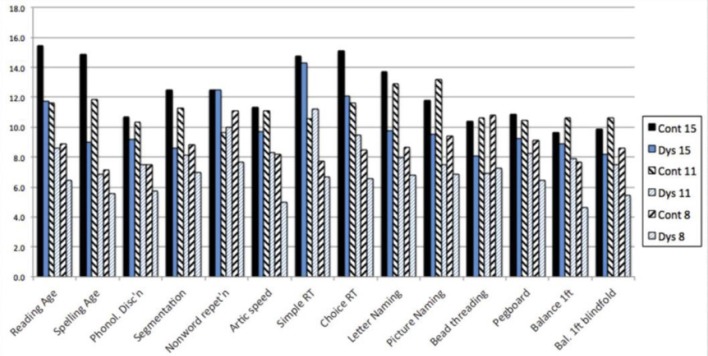
Age-equivalent scores across the range of primitive skills.

In order to facilitate comparison between tests, the results for each test have been converted to the age-equivalent scores, taking the data from our control groups together with control data from other studies where possible (Figure 2). As expected, there were severe difficulties for the phonological skills—phonological discrimination and segmentation, though not for nonsense word repetition, which is generally considered a phonology/memory task. Articulation speed was also severely delayed, significantly worse than the RA controls—indicating a disorder. Letter naming and picture naming were also significantly slower than the RA controls—indicating a disorder. Finally, inspection of the four physical coordination tasks (bead threading, pegboard, balance on one foot and balance on one foot blindfold) indicates that there is also a disorder for these, with performance worse than the RA controls. For detailed analysis, see Nicolson and Fawcett (1994).

Individual analyses indicated that the majority of the dyslexic participants had delays of at least one standard deviation against their age-matched controls on most of the basket of skills tested. There was, therefore, developmental delay on most of the skills tested. On the other hand, the dyslexic children were continuing to improve on most of these skills (cross-sectionally) and there was therefore little evidence that they would not continue to improve with maturation.

### Study 2: Extended Training on a Keyboard Game

**Figure 3:**
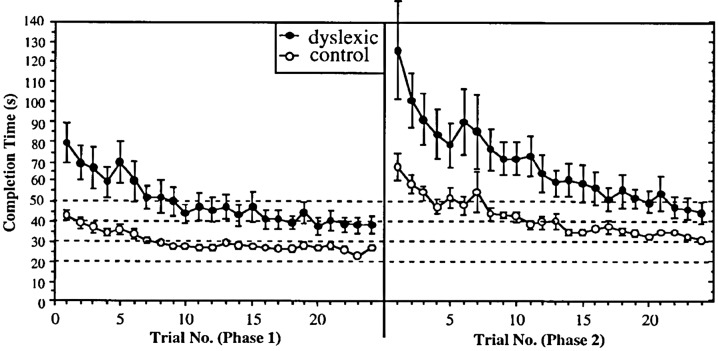
Speed of circuit completion with practice.

The above study, in common with almost all dyslexia studies, used a cross-sectional design. Such studies investigate the products of learning but not the processes. In this study (Nicolson and Fawcett, 2000), we used a longitudinal design, investigating extended learning on a keyboard-based computer game, where use of the keys moved the player around a maze, pursued by Pacman ghosts. The general results are shown in Figure 3. It may be seen that the dyslexic group were much slower initially, they took longer to reach asymptote (maximum speed) and also were slower at asymptote. However, we also investigated the effects of changing the key-finger mappings once they had automatized the initial ones, thereby forcing the participants to “unlearn” these previous mappings. We found that the dyslexic participants were actually more impaired by the change. Furthermore, we established that (after relearning the new mapping to automaticity) and then retesting 6 months later, the dyslexic participants were if anything less affected by interference while doing the task. We concluded that the dyslexic participants had equivalent “quantity” of automatic performance (as indexed by difficulty of unlearning and resistance to interference) but reduced “quality” of automatic performance (as indicated by speed and accuracy).

### Study 3: Learning Processes for Primitive Skills

**Figure 4:**
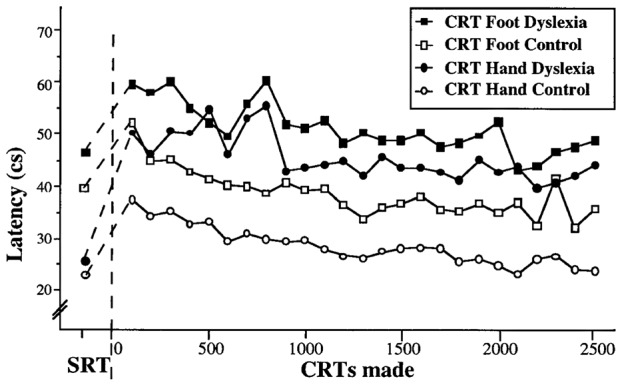
Speed of primitive skill with practice.

The above Pacman study provided a unique perspective on the learning processes in dyslexia but was subject to the limitation in interpretation that the task was complex, relying on a range of eye-hand skills that involved prior learning. Consequently, in a further study reported in Nicolson and Fawcett (2000) we investigated the learning of a “primitive skill,” namely blending a simple reaction (pressing a button on perceiving a stimulus) to a choice reaction (pressing different buttons depending on the type of stimulus). Furthermore, we investigated a familiar response (a button press by hand on hearing a tone) with a novel response (a button press by foot on seeing a flash). The results of extensive practice are shown in Figure 4.

The qualitative interpretation is clear. Both groups performed the hand press faster than the foot press. Both groups showed the standard speed deficit in moving from the simple reaction to the choice reaction. As in the Pacman study, the dyslexic participants started significantly slower and finished significantly slower. Indeed, the dyslexic participants were slower on the hand response than the non-dyslexic participants on the foot response. Furthermore, though not shown here, the dyslexic participants made significantly more errors at all stages. Consequently, even for a novel response, the dyslexic participants were markedly slower—from the start—than the non-dyslexic participants. Of particular interest, however, it is possible to fit the mean performance data using the Power Law of Practice (Newell and Rosenbloom, 1981) *P(n)* = Cn^α^ where *n* is the trial number, *P(n)* is performance on trial *n*, *C* is a constant and α is the learning parameter. This led to the following best fit equations (Nicolson and Fawcett, 2008). The best-fit curves for hand response CRT were *t* = 53.9 *n*^−0.073^ for the dyslexic participants and *t* = 39.4 *n*^−0.141^ for the typically developing participants. For the foot responses, the corresponding best-fit curves were *t* = 62.3 *n*^−0.086^; *t* = 50.4 *n*^−0.116^ respectively. The parameter *B* was higher for the dyslexic participants (around 30% on average), reflecting the slower initial performance on the CRT. Even more interesting, however, is the difference in learning rate α between groups (0.141 vs. 0.073; 0.116 vs. 0.086 for the hand and foot responses respectively). Even for the foot responses (a novel response) the learning rate of the typically achieving participants was 33% faster. This analysis leads to the “cube root rule” that, in order to achieve equivalent performance to typically achieving children, dyslexic children need exponentially longer—if a task requires eight trials, a dyslexic child will take twice as long, if it typically takes 27 trials a dyslexic child will take three times as long, if it typically takes 1,000 trials, a dyslexic child would take 10 times as long (10,000 trials).

In summary, the more complex the task, the greater the delay suffered by dyslexic children. This finding, if replicated for other skills, provides a clear explanation of why, even with best practice at support, it has proved frustratingly difficult to overcome the difficulties suffered by dyslexic children in learning to read, a skill that takes hundreds of hours to master.

## Development of Dyslexia

We believe that these studies provide a window on the development of neural networks. Prior habits are harder to unlearn, and the resulting networks work less efficiently than normal, even when given ideal conditions of consistent mapping.

### Dyslexia: The Neural Noise “Minimal Hypothesis”

In commenting on the above theories one might argue that they all (including our own) show a degree of “premature specificity,” in that they posit a framework without first ruling out all possible alternatives, and that many lack a developmental framework. In moving towards a fourth framework more powerful than our previous three of automaticity deficit, cerebellar deficit and procedural learning deficit, we take a “first principles” approach. One of the major difficulties in understanding dyslexia is that one has to undertake the “reverse engineering” approach to development in that by the time dyslexia is diagnosed, the child will be at least 6 or 7 years old and one, therefore, has to speculate about the antecedent processes. Our approach to this issue, and to avoiding premature specificity, is to introduce the simplest possible explanation for the situation at birth, then characterize how this would affect subsequent development, with the hope that this “forward model” might be able to meet up with the reverse model moving backwards in time from the known problems in early school.

Let us attempt here to build this “forward” model. First, let us take a “minimal brain dysfunction” approach where slight differences in brain organization are caused by a slightly abnormal neural migration process during gestation, which leads to slightly less well-organized cortical (and perhaps sub-cortical) “tabula rasa” in which subsequent learning takes place (Galaburda et al., 1990; Galaburda, 2005). This might involve dysplasias and ectopias as identified by Galaburda (1986), or the slightly coarser cortical columnar arrangement identified by Casanova et al. (2002). It is important to recognize that this is not necessarily a neural structure or connectivity issue. It may arise from subcellular processes such as neurotransmitter release parameters which would lead to variability in the strength and the timing of impulses within the neuron. Regardless of the specific cause, this can be characterized in the classic information processing terms of greater intrinsic processing variability, that is higher processing noise, which leads to a lower signal-to-noise ratio (Sperling et al., 2005). This may be localized within specific brain regions or it may be throughout the brain. What effects on learning and development would this simple noise framework predict? Skill development is dependent upon automatisation, which is in turn dependent upon consistency of processing and is impaired by any variability of processing (Shiffrin and Schneider, 1977). Consequently, increased noise will lead to greater variability and thus a higher quality signal or longer experience will be needed to allow the same degree of learning. This will lead to difficulty in building up automatic skills. Hence, in order to achieve average levels of performance on skills that normally-achieving children undertake automatically, dyslexic children will have to “consciously compensate,” that is, consciously monitor the skills involved, thus reducing the effective cognitive resources available.

Second, once dyslexic children have built up a habit (albeit slowly and inefficiently) they will have difficulties “unlearning” that habit—see Study 2 above. This is often a key requirement for the construction of cumulative skills such as reading, where one develops a skill through a series of stages where transition from one stage to the next requires a degree of unlearning of the previous habits. For instance, the change from phonological decoding to whole word decoding is a key stage in the transition to fluent reading, and one that is a specific difficulty for dyslexic children (Finn et al., 2014).

Third, increased variability of processing will lead also to variability in the temporal dimension, that is, timing consistency. Again, this will interfere with the development of a range of skills, especially those that involve explicit or implicit actions. In terms of cerebellar processing, the “temporal error” feedback *via* the climbing fibers will be less consistent, leading to inefficient learning, irrespective of cerebellar functionality (Sokolov et al., 2017). This includes both the explicit timing and also the implicit timing such as the proprioceptive feedback needed to undertake skilled movements or to catch a ball.

Fourth, and a key component that we had overlooked in our earlier frameworks, the intrinsic variability will preclude, or at least delay, the construction of integrated neural networks, such as the fluent reading network, the balance network, the executive function networks, and the default mode networks that characterize the information-processing of older children and adults.

In short, automatic processing, including all forms of implicit learning, would be impaired. By contrast, declarative processing—broadly, processing knowledge that can be retrieved and verbalized—would be unimpaired and possibly over-performing.

### Neural Commitment and Infant Speech Development

One of the abiding challenges of dyslexia research is to explain why it is that dyslexic children successfully achieve the extremely challenging task of the initial acquisition of speech and language and yet they fail catastrophically in the related, and superficially simpler, task of learning to read. Given our developmental approach, it is therefore important to consider the developmental processes involved in infant language learning. There is now a growing consensus as to the processes involved in learning to speak—knowledge that was not available when the phonological deficit hypothesis was first proposed. This analysis (Kuhl, 2004) and the follow-up analyses (Meltzoff et al., 2009; Kuhl, 2010) represent the current understanding on how speech and language develop over the first year of life.

Kuhl and her colleagues considered two dimensions: receptive (speech perception) and expressive (speech production). In terms of perception, in its first 6 months any normally developing infant can, in principle, discriminate any of the sounds in any of the human languages. However, in months 6–12, the infant becomes a specialist in its mother tongue, essentially using statistical learning to identify the regularities of its heard environment. This leads to good discrimination of the phonemes in its own language, but at the expense of phonemes in other languages. The classic example is the fact that Japanese infants can discriminate /l/ from /r/ at 6 months but lose this ability by 12 months, because the distinction is of no significance in the Japanese language.

The key learning process here is statistical (self-organizing) learning, which “tunes” the hearing of the infant to their own mother tongue phonology and prosody. Mere exposure to speech in its many forms and speakers allows the infant’s auditory processing to “neurally commit” to the phonemes that it encounters, and to classify the different sounds into the appropriate phoneme categories. A range of additional natural learning abilities is also involved including trial-and-error learning to speak the phonemes, and social learning to interact with the mother. These factors—all general purpose learning capabilities, but scaffolded by personalized interaction with the mother—provide significant advantages for speech production and perception (Meltzoff et al., 2009).

Kuhl (2004, p.831) introduced the term “neural commitment” as the third mechanisms by which infants learn their first language. She defined it as follows: “*learning results in a commitment of the brain’s neural networks to the patterns of variation that describe a particular language. This learning promotes further learning of patterns that conform to those initially learned, while interfering with the learning of patterns that do not conform to those initially learned*.” Finally, as the language-specific hearing and speaking skills develop, the underlying neural circuits “commit” to that processing method. There is no going back.

Neural commitment is, in our view, the key underlying principle. It is particularly clear in the case of language. Once an English-hearing infant commits to the English phonological system, he or she can no longer hear the distinctions made in different language systems. That is the hearing and speaking neural networks in the ear and mouth inwards have developed so as to ignore non-relevant language information to the extent that is not accessible to processing at all. We have constructed signal-processing capabilities that are outside conscious control or inspection, and, critically, are almost impossible to unlearn. Prior experience in learning has built structures that constrain and channel subsequent learning.

Interestingly, infants exposed to a bilingual language environment from birth do show some delay in their speech (Garcia-Sierra et al., 2011), a consequence of their more complex linguistic environment. Of course, the key difference from dyslexia is that the noisier internal environment of the bilingual infant is attributable to external signal complexity, and will in due course be fully resolved by the creation of a “dual language processing architecture” which essentially eliminates the noise from the signal. Indeed, for the bilingual infant this delay in neural commitment to their own mother tongues leads to extensive advantages throughout later life, with better executive function at 2 years, better working memory at 5 years (especially when the task is difficult), better perspective taking at 8 years and even protection against Alzheimer’s at 60 years (Poulin-Dubois et al., 2011; Bialystok et al., 2012; Greenberg et al., 2013; Morales et al., 2013).

Overall, it is reasonable to assume that the delays attributable to the increased internal noise of the dyslexic infant in the acquisition of language and speech would also be relatively minor. By contrast, however, the articulatory and phonological processing architecture would remain relatively inefficient. It may be relevant here that the major skills required for the initial acquisition of language—statistical sensory learning, and social learning—can be scaffolded by cerebral cortex learning processes. By contrast, articulation, imitation and hence phonological processing require the error-based learning processes scaffolded only by the cerebellum.

So, having learned to talk (and walk, which is beyond the scope this article) what next?

### Development of Executive Function

The classic Piagetian developmental framework highlighted the extraordinary cognitive development of the child from 1 to 6 years of age, moving from the sensorimotor stage through the pre-operational stage to the start of concrete operations. It is important to recognize that this development occurs in fits and starts, in different ways for different children and involves the construction of new and better ways of processing information. Of course, in terms of networks, the child is slowly constructing the networks needed to take control of the stimulus-driven automatic systems.

The Piagetian framework has been substantially replaced by the information processing framework relating to “executive function” (EF), but these are different perspectives on essentially the same process of the emergence of controlled processing. In her recent review of executive function and its development, Diamond (2013) identified three core EFs—inhibition [inhibitory control, including self-control (behavioral inhibition) and interference control (selective attention and cognitive inhibition)], working memory, and cognitive flexibility (mental flexibility, or mental set shifting and closely linked to creativity). These may be considered as the top-down processes that operate in affectively neutral contexts (“cool EF”). It is also important to note there are also “hot EF” processes—the top-down processes needed for control of anger, aggression, impulsivity and anxiety—that occur in motivationally and emotionally significant situations (Zelazo and Carlson, 2012).

Executive function develops with experience and maturity, and recent analyses (Bauer and Zelazo, 2014) reveal a consistent overall improvement throughout childhood and adolescence. Current views suggest that at 3 years of age, EFs are relatively uniform, but they differentiate into the three cool EFs and the hot EFs over the next few years. Consequently, of particular significance here is the recent literature on the development of executive function from 3 to 6 years, and in particular the role of executive functions in “school readiness” (Fitzpatrick et al., 2014). An early review (Blair, 2002) highlighted the importance not only of the “cool EF” capabilities described above but also the emotional and social EF requirements for school readiness.

### Delayed Neural Commitment and Dyslexia

We have already implicitly presented the DNC framework in the above analyses. It is nonetheless valuable to provide a clear statement. Dyslexia is associated with minimal brain differences, arising from gestation, that may be characterized as leading to increased noise in the neural circuits associated with hearing and speech (and maybe more widely). It is, of course, likely that the increased noise will be localized to distinct brain regions in different individuals, and this will lead to specific forms of processing and learning deficit, but for the present purposes, we consider a completely general formulation.

#### Implications of Delayed Neural Commitment

The framework is consistent with a range of established findings.

First, acquisition of language-related skills is delayed and less precise. This leads to less well-organized phonological networks, and delays in the construction of the “phonological module,” which leads to phonological impairments and delays. This is, of course, a starting point for any adequate theory of dyslexia. These findings are well known.

Second, more generally, the intrinsic variability in processing leads to difficulties in automatizing skills in many domains, especially those that involve language or articulation, either explicitly or implicitly. This is essentially a re-description of the automatisation deficit hypothesis, and there is extensive evidence supporting it, including evidence that dyslexic infants do indeed have less well-developed speech sound discrimination at birth (Molfese, 2000; Guttorm et al., 2005). It is also directly consistent with the data presented in Figure 2.

Third, DNC also applies to the unlearning of more primitive habits which form a valuable “scaffold” for future learning but subsequently interfere with the new system. This applies to many developmental transitions: the primitive reflexes are necessary for early survival but then interfere with subsequent skill development, the primitive sensorimotor skills that scaffold the development of Piaget’s pre-operational stage, the ability to switch attention in response to a new stimulus (which interferes with the ability to remain “on task”), and the self-determination skills that allow the 3 year old child to focus on his or her goals at the expense of other goals, but which interfere with the ability to decenter. There is evidence that significant numbers of dyslexic children have not unlearned the primitive reflexes (McPhillips et al., 2000), and, of course, the study shown in Figure 3 provides a graphic illustration of the difficulties of unlearning. There is also extensive evidence that dyslexic children, even those with extensive phonological training, do have almost insuperable difficulties in moving from the stage of word decoding to the fluent, whole word reading stage (NICHD, 2000). This suggests that they do have difficulties unlearning the phonological scaffolding stage.

Fourth, it becomes more difficult to construct the higher level neural networks needed for executive control and, later for reading. There is relatively little information available regarding the development of executive function in dyslexic children pre-school, though findings implicating executive function are now coming through (Clark et al., 2014; Varvara et al., 2014; Moura et al., 2015). There is extensive evidence that dyslexic children do not normally develop the mature “Visual Word Form Area” fluent reading networks (Shaywitz et al., 2007) or, if they do, the representations tend to be at the whole word level rather than also sub-lexically (van der Mark et al., 2009).

Fifth, more generally, the dyslexic information-processing system is more noise tolerant that is, it is less dependent upon high accuracy or consistency of signal. This is the only aspect that does not represent an overt deficit. As noted earlier, there is suggestive empirical evidence of declarative advantage in dyslexia (Hedenius et al., 2013). There is also an extensive though contested literature consistent with declarative strengths in dyslexia (Geschwind, 1982; West, 2009).

#### Distinctive Contributions of Delayed Neural Commitment

One might consider that, given its lineage in our three earlier theories, DNC does not represent a significant step forward. However, though superficially relatively modest, the reformulation brings major changes in perspective. First, it opens a direct link to current theories of the cognitive neuroscience of language and language development—a major issue for the understanding of phonological development.

Second, unlike all other formulations, it extends the discussion to consider the entire information processing architecture—the Piagetian levels, the executive functions, the working memory, the ability to inhibit natural impulses, the ability to learn by being told. It encourages the consideration of the “big picture” for human development rather than a series of independent skills.

Third, unlike our other frameworks, which were all framed in terms of deficit, DNC is value-free with regard to advantage and disadvantage, DNC leads to drawbacks in some areas but can lead to advantages in others, especially in circumstances where it is useful to maintain earlier skills, or valuable to combine two different skills which do not normally occur within the same “time window.” It also has the characteristic of biasing dyslexic people to specialize in using their declarative processing systems rather than their habit-based procedural system. Extensive use of the declarative system will lead to increasing expertise in its use, which can lead to a range of benefits in the chosen area of specialization.

#### Delayed Neural Commitment and Theories for Dyslexia

We may now return to the theories we outlined earlier. We mentioned Phonological Deficit, Double Deficit, Phonological Access, Speech rhythm deficit, visual processing deficit, auditory temporal deficit, automatisation deficit, magnocellular deficit, cerebellar deficit, and procedural learning deficit.

It may be seen either as a strength or a weakness of DNC that it provides a natural and immediate explanation for all of these frameworks, being able to bring in both the learning framework and a temporal accuracy perspective. It also provides a natural explanation of the inefficiencies in phonological access and executive function speed that are the distinctive features of the phonological access framework.

#### Delayed Neural Commitment—Re-uniting the Developmental Disorders!?

In our procedural learning deficit framework (Nicolson and Fawcett, 2007), we put forward the hope that moving focus to a network level provided the opportunity to “reunite” the developmental disorders, allowing a focus on commonalities as well as differences. The DNC framework provides a further opportunity for progress in these aims. DNC is not a dyslexia-exclusive description. It could well apply to a whole range of developmental disabilities, and will also apply to so-called typically developing children for specific aspects of their development. Therefore, DNC provides a bridge between dyslexia, other learning difficulties, and normal development.

The framework is also directly consistent with research on comorbidities. The major comorbidities for dyslexia are with DCD, SLI, and attention deficit disorder (ADHD). One might expect that the overlap with DCD would reflect the motor component (rather than the language component) of the procedural learning networks, though the theoretical development of DCD appears to be less advanced that of dyslexia. Suggestive evidence of cerebellar-type problems in DCD arise from a prism adaptation study (Brookes et al., 2007) and also a recent finding of the impact of task difficulty on motor performance in DCD which is equivalent to the automatisation deficit account (Cantin et al., 2014). Interestingly, there is also building evidence of EF problems in DCD (Rahimi-Golkhandan et al., 2014; Tal Saban et al., 2014).

The role of EF problems in ADHD is long established (Barkley, 1997; Willcutt et al., 2005) with a key issue being inhibitory control, the ability to withstand the urge to make the automatic response, though Barkley also highlights the issue of speech internalization. Furthermore, the incidence of motor skill problems in ADHD was highlighted by Kaiser et al. (2015), which concluded that “*More than half of the children with ADHD have difficulties with gross and fine motor skills. The children with ADHD inattentive subtype seem to present more impairment of fine motor skills, slow reaction time, and online motor control during complex tasks*” (p.338).

There is a strong (but not bidirectional) relationship between SLI and dyslexia, with many children with SLI having reading problems. There is clear evidence of procedural learning problems in SLI (Ullman and Pierpont, 2005; Lum et al., 2014) and there is again clear evidence of impaired executive function (Im-Bolter et al., 2006; Henry et al., 2012).

In short, the comorbidities between all four learning disabilities are naturally accounted for within a DNC framework in which each learning disability has a cluster of problems relating to motor skill, executive function, and language, all consistent with difficulties in some or all components of the procedural learning system.

### Delayed Neural Commitment and Support for Dyslexia

Traditional good practice for supporting dyslexic children (Gillingham and Stillman, 1960; Hulme, 1981; Miles, 1989; Hickey, 1992) requires that for dyslexic children an exceptionally structured, explicit, systematic, and comprehensive intervention approach is needed, progressing in a series of small steps, with each step mastered before the next one is introduced. More recently there have been major government efforts to develop phonics-based support to ensure that early readers start reading instruction with the phonological skills needed to benefit from systematic phonics-based teaching (Lonigan and Shanahan, 2009; Rose, 2009). Interestingly, a major meta-analysis of the decades of research into approaches to reading instruction (Stuebing et al., 2008) concluded that the key to successful reading instruction was more down its systematicity than to its strategy, and that systematic phonics-based effects were beneficial, but so were systematic non-phonic based systems. The results of the studies reported here are therefore consistent with accepted good practice in support for dyslexic children, but advance considerably the underlying theoretical rationale, as we discuss below.

**Figure 5:**
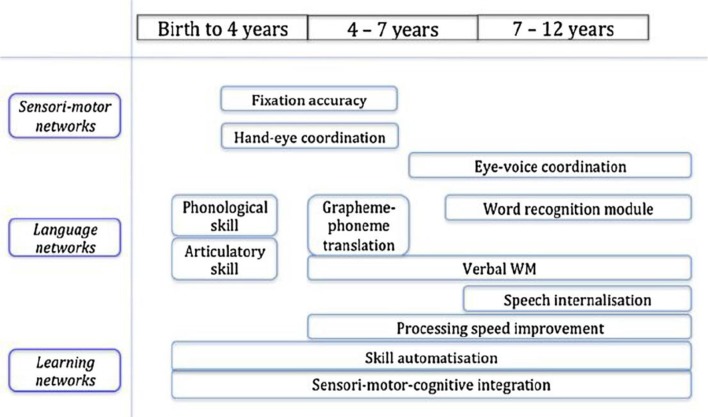
Proposed components relating to the development of reading (Mariën et al., 2014).

Reading is a complex skill, in that the complete skill depends upon a range of sub-skills (see Figure 5 for an illustrative example of the multiple underlying skills, networks and their developmental trajectory). It is clear that for fluent performance of the complete skill the sub-skills should be automatized, but one important question is whether each sub-skill should be automatized individually, in isolation (which is easier), or whether all the sub-skills need to be automatized in the context of performing the complete skill. In the case of physical skills (Shea and Morgan, 1979) established that it is important not to train the sub-skills purely in isolation. If this happens there is a danger that the automatic method that the subject develops for the sub-skill might require some resources that are needed for performance of one of the other sub-skills, and so, when one attempts to blend the sub-skills into the complete skill, there is interference between the sub-skills, preventing the complete skill from being performed efficiently. Therefore, in order to make sure that this interference will not arise, it is important to interleave sessions of the complete skill with automatisation training on the sub-skills, so that the sub-skills are learned in a compatible fashion. This approach is sometimes known as that of “whole-part-whole” task training (Swanson and Law, 1993).

This approach has been established as more effective for individual skills, but our contention is that the situation is, in fact, more critical for skills that develop over several years, and are in fact underpinned by the creation of neural networks. Consider the endpoint of fluent reading, which involves an extended network including the VWFA that links into and subsumes the earlier reading networks for phonology, letter fixation, semantics and single word reading. The danger is that if any of these skills (such as phonology) is developed in advance of the other skills (and before the appropriate attentional networks have been created), then progression from the letter-by-letter reading stage to the whole word reading stage will be impeded (and perhaps impossible) because there will be no linkage between the networks involved, and the word stimulus will be “captured” by the more primitive circuitry, in an analogous fashion to the intractability of acquiring full second language fluency later in life.

This analysis makes it clear that practice alone is not sufficient, that systematic practice alone on sub-skills is not sufficient, and even that whole-part-whole practice on sub-skills is not sufficient. A key further requirement is that the necessary neural networks are available to allow the sub-skills to be integrated into a fluent process. This developmental/maturational framework has major implications for the pedagogic approaches for children with developing executive skills and has clear resonance with Piaget’s framework (Inhelder and Piaget, 1958) and for the concepts of classroom readiness (Duncan et al., 2007) and reading readiness (Petty, 1939).

The DNC framework makes it very clear that an effective support system needs to consider not only the state of development of a child’s reading and pre-reading skills (reading readiness) but also the state of the child’s executive function skills. There has been considerable, and successful recent research on interventions designed to improve executive function pre-school (Diamond, 2013; Fitzpatrick et al., 2014). These are mostly on children from disadvantaged backgrounds, but we consider that the findings are of general applicability.

Finally, and of the utmost importance, the framework highlights the significant risks attached to well-meant initiatives aimed at pre-reading interventions for dyslexia. The whole-part-whole framework highlights the dangers of premature functional specialization, such that if a sub-skill is taught to automaticity outside the intended “whole skill” context, it becomes impossible subsequently to integrate the sub-skill within the whole skill—akin to our analyses of unlearning. While this remains speculative at this stage, one would expect that similar limitations occur at the level of neural networks, and that it is important to ensure that the appropriate networks have been created before teaching sub-skills.

Given the delays in skill development and neural network development under DNC (even if not so stark as suggested by the cube root rule), it is important to ensure that all the executive function and pre-reading skills are in place together at the start of formal reading instruction. Consequently, it may well be that delaying the start of formal reading instruction by assuring that the classroom readiness and reading readiness skills are in place will prove to be a more effective strategy that the current attempt to accelerate learning in specific sub-skills in isolation.

### Limitations of the Delayed Neural Commitment Framework

The DNC framework is (designedly) very broad. The fact that it has clear pedagogic implications despite this breadth is therefore particularly striking. Three major criticisms may be leveled at the framework, under-specificity, lack of focus and lack of direct evidence. All three are fully justified, but not intrinsically damaging, rather reflecting the opportunity for fruitful further research.

Under-specificity is, of course, a key issue. We have not specified the cause of the increased neural noise, and this may indeed arise for a range of reasons—genetic predisposition to abnormal development in (specific) brain regions, gestational insults such as caused in fetal alcohol syndrome or high levels of maternal stress, peri-natal difficulties, or post-natal issues such as impoverished environment, otitis media (glue ear), childhood stress, and so on. All these are likely to lead eventually to reading problems.

It is likely that different children will have increased neural noise in specific brain regions (and hence DNC in specific skills) as a consequence. In our view, identification of the specific difficulties leading to development differences is a key requirement of early diagnosis, and that such a diagnosis should lead to an appropriately targeted intervention. This is a clear target for pedagogical research. A key insight of DNC is that the (development of) neural networks involved may well be the appropriate level of analysis both for diagnosis and for support.

Lack of direct evidence is an important issue. Here, we have provided a range of suggestive but indirect evidence. The newly developed tools in terms of analyses of intrinsic connectivity and of EEG recordings do provide, for the first time, the opportunity to undertake objective analyses of the neural noise within developing neural systems for individual infants and children. This, therefore, holds out the promise of developing a truly individualized analysis-and-support system to facilitate unprecedently effective skill and network development. Again, this is a clear target for pedagogical cognitive neuroscientific research.

## Conclusion

In this article, we have explored the issue of the development of dyslexia. In the interests of avoiding premature theoretical commitment, we adopted the least committal form of theory possible, in terms of increased neural noise. We explored its likely effects on the developmental processes. We established that a major factor would be DNC, both for individual skills and for the development of integrative neural networks. DNC explains not only the delay in development of specific skills and of automaticity but also delays both in constructing new neural circuits (as required for executive function and for internalized speech) and in bypassing or eliminating the previous, less efficient neural circuits. The framework is directly compatible with the major theories of dyslexia and provides a natural explanation of the comorbidities between dyslexia and other learning disabilities. Furthermore, it has major implications for educational neuroscience and for educational practice, both for reading and for pre-school education. This model could inform education professionals on the need for a broader approach to dyslexia, encompassing elements such as speed and automaticity and other aspects of executive function, in addition to the well-supported phonological approach.

## Ethics Statement

University of Sheffield ethics committee provided ethics permission for this series of studies, full written informed consent procedure was used in controlled studies, our panel of subjects received support and guidance over several years, and anonymity was maintained for children with dyslexia.

## Author Contributions

In terms of contributions to the research team, both RN and AF contributed to the design, execution and interpretation of the studies. RN led on preparing this article but both authors were involved in contributing to the article and reviewing the literature in the article presented here.

## Conflict of Interest Statement

The authors declare that the research was conducted in the absence of any commercial or financial relationships that could be construed as a potential conflict of interest.
